# Insulin resistance is significantly associated with the metabolic syndrome, but not with sonographically proven peripheral arterial disease

**DOI:** 10.1186/1475-2840-12-106

**Published:** 2013-07-17

**Authors:** Alexander Vonbank, Christoph H Saely, Philipp Rein, Heinz Drexel

**Affiliations:** 1 Vorarlberg Institute for Vascular Investigation and Treatment (VIVIT), Feldkirch, Austria; 2 Department of Medicine and Cardiology, Academic Teaching Hospital Feldkirch, Feldkirch, Austria; 3 Private University of the Principality of Liechtenstein, Triesen, Liechtenstein; 4 Drexel University College of Medicine, Philadelphia, PA, USA

**Keywords:** HOMA index, Atherothrombosis, Atherosclerosis, Insulin, Metabolic disorder

## Abstract

**Objective:**

Insulin resistance (IR) is the key feature of the metabolic syndrome (MetS); its association with peripheral arterial disease (PAD) is unclear. We hypothesized that IR is associated with both the MetS and sonographically proven PAD.

**Methods:**

IR was determined by the Homeostasis Model Assessment (HOMA) index in 214 patients with sonographically proven PAD as well as in 197 controls, who did not have a history of PAD and in whom coronary artery disease was ruled out angiographically; the MetS was defined according to NCEP-ATPIII criteria.

**Results:**

HOMA IR scores were significantly higher in MetS patients than in subjects without the MetS (5.9 ± 6.2 vs. 2.9 ± 3.9; p <0.001). However, HOMA IR did not differ significantly between patients with PAD and controls (4.2 ± 5.4 vs. 3.3 ± 4.3; p = 0.124). When both, the presence of MetS and of PAD were considered, HOMA IR was significantly higher in patients with the MetS both among those with PAD (6.1 ± 5.7 vs. 3.6 ± 5.2; p<0.001) and among controls (5.8 ± 6.8 vs. 2.3 ± 1.8; p <0.001), whereas it did not differ significantly between patients with PAD and controls among patients with the MetS (5.8 ± 6.8 vs. 6.1 ± 5.7; p = 0.587) nor among those without the MetS (2.3 ± 1.8 vs. 3.6 ± 5.2; p = 0.165). Similar results were obtained with the International Diabetes Federation definition of the MetS.

**Conclusion:**

IR is significantly associated with the MetS but not with sonographically proven PAD.

## Introduction

The metabolic syndrome (MetS), a cluster of cardiovascular risk factors including central adiposity, hypertension, dyslipidemia and impaired glucose metabolism has been consistently associated with an increase in the incidence of coronary heart disease, stroke, and cardiovascular mortality [1-7].

Pathophysiologically, insulin resistance is considered the key feature of the MetS [8]. Indeed, insulin resistance is associated with all component features of the MetS [9-12]. In epidemiological studies, insulin resistance typically is quantified by the Homeostasis Model Assessment (HOMA) index. We could previously show that HOMA insulin resistance is associated with the MetS but not with coronary artery disease (CAD) [13].

Peripheral arterial disease (PAD) is another important manifestation of systemic atherosclerosis which confers significant cardiovascular morbidity and mortality [14]. Indeed, the prognosis of PAD patients in general is worse to that of CAD patients [15]. However, risk factors for PAD have not been as thoroughly investigated as risk factors for CAD.

Whereas type 2 diabetes is a well established major risk factor for PAD [16], only very limited data available on the association between the MetS and PAD [17-21]. In particular, the role of insulin resistance in PAD is unclear. Because PAD causes skeleton muscle ischemia, the ischemic muscle could be a link to insulin resistance [22]. Thus PAD potentially could induce muscular insulin resistance.

In the present study, we therefore determined HOMA insulin resistance in a cohort of sonographically characterized PAD patients and in controls without signs or symptoms of PAD, in whom in addition CAD was ruled out angiographically. We hypothesized that insulin resistance is associated with both sonographically determined PAD and with the MetS.

## Patients and methods

### Study subjects

From August 2007 through December 2010 we enrolled 214 consecutive Caucasian patients with intermittent claudication who were referred for the evaluation of established or suspected PAD to the Angiology Clinic at the Academic Teaching Hospital Feldkirch, a tertiary care centre in western Austria (state of Vorarlberg). This is the only angiologic clinic in Vorarlberg; patients typically are referred to there by general practitioners or specialists in internal medicine. Assessments regarding the diagnosis of PAD were part of the common clinical examination; metabolic assessments including the measurement of insulin resistance were part of the study protocol. Patients were instructed to fast overnight when they arranged their appointment to the angiologic clinic.

As controls, we used a cohort of 197 patients in whom CAD was ruled out angiographically and who had no PAD. Both PAD patients and controls were enrolled in a consecutive manner. Patients with type 1 diabetes were not enrolled; no other exclusion criteria did apply. The Ethics Committee of the University of Innsbruck approved the present study, and all participants gave written informed consent.

Information on conventional vascular risk factors was obtained by a standardized interview; and systolic/diastolic blood pressure was measured by the Riva–Rocci method under resting conditions in a sitting position at the day of hospital entry at least 5 h after the hospitalization for planned coronary angiography in our control group and prior to the angiologic examination in our patients with PAD. Hypertension was defined according to the Seventh Report of the Joint National Committee on Prevention, Detection, Evaluation, and Treatment of High Blood Pressure [23], and type 2 diabetes mellitus (T2DM) was diagnosed according to World Health Organization criteria [24]. Height and weight were recorded, and body mass index (BMI) was calculated as body weight (kg)/height (m)^2^. Table 1 shows pre-existing medication in PAD cases and in controls.

**Table 1:** Medication use in patients with peripheral artery disease and controls

	**PAD (n = 214)**	**Controls (n = 197)**	**p-value**
Aspirin (%)	65.9	54.3	0.005
Clopidogrel (%)	29.0	4.9	<0.001
Metformin (%) *	18.4	28.9	<0.001
Sulfonyrea (%) *	13.8	17.8	0.775
Glitazone (%) *	1.0	1.4	0.689
Insulin (%) *	7.9	5.1	0.211
Statins (%)	47.2	26.4	<0.001
Angiotensin Converting Enzyme Inhibitors (%)	42.5	23.4	<0.001
Calcium Antagonists (%)	31.1	9.1	<0.001
Beta Adrenoreceptor Blocking Agents (%)	37.9	44.7	0.161
Angiotensin II Receptor Blocking Agents (%)	11.2	6.1	0.067

PAD denotes peripheral artery disease.

* Percentage of subjects with type 2 diabetes (n = 87 and 45 in the PAD and in the control group, respectively).

According to National Cholesterol Education Programme ATP-III criteria [25] (NCEP – ATPIII), the MetS was diagnosed in the presence of any three of: waist circumference >102 cm in men and >88 cm in women, triglycerides ≥150 mg/dl (1.7 mmol/l), high density lipoprotein (HDL) cholesterol <40 mg/dl (1.0 mmol/l) in men and <50 mg/dl (1.3 mmol/l) in women, blood pressure ≥130/ ≥85 mmHg, or fasting glucose ≥100 mg/dl (5.6 mmol/l). Using International Diabetes Federation (IDF) criteria [26], the MetS was diagnosed in patients who had a large waist circumference (≥94 cm in men and ≥80 cm in women) plus any two of: triglycerides ≥150 mg/dl (1.7 mmol/l) or specific treatment for this lipid abnormality, HDL cholesterol <40 mg/d (1.0 mmol/l) in males and <50 mg/dl (1.3 mmol/l) in females or specific treatment for this lipid abnormality, systolic blood pressure ≥130 or diastolic blood pressure ≥85 mmHg or treatment of previously diagnosed hypertension, and fasting plasma glucose ≥100 mg/dl (5.6 mmol/l) or previously diagnosed T2DM.

For Ultrasound examination we used a Philips iU22 ultrasound system. PAD was diagnosed by direct visualization of atherosclerotic plaques in peripheral arteries of the lower limbs. The scanning protocol included a completed lower limb sonography (we scanned bilateterally the Aa. illiaca ext., Aa. fem. com., Aa. fem. sup., Aa. fem. prof., Aa. popl., Aa. tib. ant., Aa. tib. post. and Aa. Brach., respectively). PAD was defined as any sonographically detectable atherosclerosis in peripheral arteries [27]. All patients in the PAD group had at least one stenosis of more than 50% of at least one of these arteries. Coronary angiography in controls was performed with the Judkin's technique. Coronary arteries were classified as normal in the absence of any visible lumen narrowing at angiography [28].

For the evaluation of ankle brachial index (ABI) systolic pressure was measured in the supine position in the right arm (brachial artery) and in the posterior tibial artery of both ankles with an 8-MHz Doppler probe. The ABI was calculated by dividing the systolic blood pressure in the ankle by the systolic blood pressure in the arm.

### Laboratory analyses

Venous blood samples were collected after an overnight fast of 12 h before ultrasound was performed, and laboratory measurements were performed from fresh serum samples, as described previously [29]. Patients with diabetes did not take their antidiabetic oral medication before the blood sample was drawn. Oral glucose tolerance tests were performed and postchallenge glucose was measured as plasma glucose at 2 hours after an oral 75 gram glucose load in individuals without known diabetes. Serum triglycerides, total cholesterol, low density lipoprotein (LDL) cholesterol, HDL cholesterol, apolipoprotein B, apolipoprotein A1, CRP, and plasma glucose were determined on a Cobas Integra 800® (Roche, Basel, Switzerland). Haemoglobin A1c (HbA1c) was determined by high-performance liquid chromatography on an ADAMS A1c HA-8160® (Menarini, Florenz, Italy). Plasma insulin was measured with a Roche Cobas E601® (Roche, Basel, Switzerland). HOMA index was calculated by the formula fasting insulin [μU/ml] × fasting glucose [mg/dl] / 405 [30].

### Statistical analysis

Differences in patient characteristics were tested for statistical significance with the Chi square test for categorical variables; the Mann–Whitney–U and Kruskal–Wallis tests were used for continuous variables, as appropriate. Spearman rank correlation coefficients were calculated. To test for independent determinants of continuous variables, analysis of covariance (ANCOVA) was performed, using a general linear model approach. Results are given as mean ± standard deviation if not denoted otherwise. Two-sided p-values <0.05 were considered significant. Sample size calculations showed that assuming a standard deviation of 1.5 times the population mean, 190 patients would be needed per study group to detect a between–group difference of HOMA insulin resistance scores of 20% with a power of 80% at an alpha-fault of 0.05. Statistical analyses were performed with the software package SPSS 16.0 for Windows (SPSS, Inc., Chicago, IL).

## Results

### Patient characteristics

Among our 214 PAD patients, there was a preponderance of male gender (73.4%), and a very high prevalence of T2DM (40.7%), hypertension (87.9%), and smoking (84.6%). From our PAD patients, 53.8% had an ABI ≤0.9 and 46.2% had an ABI ≤0.5. In the control group (n = 197), there was a preponderance of female gender (62.9%), as well as a lower prevalence of type 2 diabetes (22.8%), hypertension (74.6%), and smoking (50.3%) when compared to the PAD patients.

Overall, 112 (27.3%) subjects had the MetS as defined by NCEP-ATP-III criteria; considering both the presence of the MetS (NCEP-ATP-III criteria) and the presence of PAD, 142 subjects had neither the MetS (ATP-III definition) nor sonographically proven PAD, 55 had the MetS, but not sonographically proven PAD, 157 did not have the MetS but had sonographically proven PAD, and 57 had both, the MetS and sonographically proven PAD. Table 2 summarizes patient characteristics in these four groups. Table 1 shows medication use in patients with PAD and in control subjects and Table 3 shows medication use in the 4 patient subgroups considering both the presence of PAD and of the MetS.

**Table 2:** Patient characteristics in subgroups with respect to both the presence of peripheral artery disease and the presence of the metabolic syndrome

	**PAD+/MetS+ (n = 57)**	**PAD+/MetS- (n = 157)**	**PAD-/MetS+ (n = 55)**	**PAD-/MetS- (n = 142)**	**p-value**
Age (years)	64.9 ± 10	67.5 ± 10	62.7 ± 12	60.8 ± 10	<0.001
Male gender (%)	70.2	74.5	34.5	38.0	<0.001
BMI (kg/m^2^)	30.1 ± 5	25.9 ± 4	27.7 ± 4	28.8 ± 6	<0.001
Waist-to-hip ratio	1.0 ± 0.8	0.9 ± 0.8	1.0 ± 0.6	0.9 ± 0.7	<0.001
Waist circumference (m)	108.2 ± 11	97.9 ± 12	107.9 ± 10	95.3 ± 13	<0.001
Hypertension (%)	96.5	78.3	52.7	59.9	<0.001
Smoking (%)	84.2	84.7	47.3	51.4	<0.001
Type 2 Diabetes (%)	68.4	30.6	40.0	16.2	<0.001
Total cholesterol (mg/dl)	180.6 ± 46	180.1 ± 41	197.6 ± 50	202.9 ± 45	<0.001
LDL cholesterol (mg/dl)	107.6 ± 41	109.6 ± 37	133.7 ± 42	131.9 ± 39	<0.001
HDL cholesterol (mg/dl)	44.5 ± 15	54.5 ± 16	49.8 ± 15	64.5 ± 20	<0.001
Triglycerides (mg/dl)	218.4 ± 142	129.1 ± 79	197.5 ± 99	115.7 ± 70	<0.001
Fasting glucose (mg/dl)	144.6 ± 68	103.4 ± 27	133.9 ± 61	96.7 ± 22	<0.001
Fasting insulin (μU/ml)	17.1 ± 11	13.1 ± 16	16.9 ±13	9.3 ± 6	<0.001
Postchallenge glucose (mg/dl)	205.1 ± 94	132.9 ± 53	113.5 ± 53	130.3 ± 72	<0.001
HbA1c (DCCT) (%)	7.3 ± 2	5.9 ± 0.8	6.6 ± 2	5.7 ± 0.7	<0.001
HbA1c (IFCC) (mmol/mol)	56 ± 2	41 ± 15	49 ± 2	39 ± 16	<0.001
Apolipoprotein A1 (mg/dl)	147.3 ± 34	158.1 ± 35	146.5 ± 26	164.7 ± 32	<0.001
Apolipoprotein B (mg/dl)	85.5 ± 11	77.6 ± 21	92.3 ± 26	82.8 ± 22	<0.001
CRP (mg/dl)	1.3 ± 3	0.9 ± 2	0.4 ± 0.5	0.3 ± 0.5	<0.001
Leukocytes (10^9^/l)	7.3 ± 2	7.4 ± 2	6.4 ± 2	6.6 ± 2	0.001
Systolic blood pressure (mmHg)	151.1 ± 21	140.0 ± 21	140.5 ± 14	131.1 ± 19	<0.001
Diastolic blood pressure (mmHg)	84.3 ± 11	78.2 ± 12	86.1 ± 8	80.3 ± 9	<0.001

BMI denotes body mass index, LDL low density lipoprotein, HDL high density lipoprotein, MetS metabolic syndrome and PAD peripheral artery disease; postchallenge glucose is plasma glucose at 2 h after an oral 75 g glucose load. To convert values for fasting plasma glucose to mmol/l multiply by 0.0555, to convert values for triglycerides to mmol/l multiply by 0.0113 and to convert total cholesterol, LDL cholesterol, or HDL cholesterol to mmol/l multiply by 0.0259; p-values are given for the all overall difference between study groups.

**Table 3:** Medication use in subgroups with respect to both the presence of peripheral artery disease and the presence of the metabolic syndrome

	**PAD+/MetS+ (n = 57)**	**PAD+/MetS- (n = 157)**	**PAD-/MetS+ (n = 55)**	**PAD-/MetS- (n = 142)**	**p-value**
Aspirin (%)	67.9	67.8	58.2	52.8	0.006
Clopidogrel (%)	26.8	31.1	3.6	4.9	<0.001
Metformin (%) *	30.3	5.1	9.1	5.6	0.007
Sulfonyrea (%) *	24.2	3.4	3.6	5.6	0.050
Glitazone (%) *	6.1	0.9	0.0	0.0	0.011
Insulin (%) *	15.2	10.3	3.6	5.6	0.040
Statins (%)	75.0	69.2	21.8	28.2	0.003
Angiotensin Converting Enzyme Inhibitors (%)	49.1	40.1	24.5	23.9	<0.001
Calcium Antagonists (%)	36.8	29.3	10.9	8.5	<0.001
Beta Adrenoreceptor Blocking Agents (%)	31.6	40.1	61.8	38.0	0.459
Angiotensin II Receptor Blocking Agents (%)	12.3	10.8	9.1	4.9	0.042

PAD denotes peripheral artery disease.

* Percentage of subjects with type 2 diabetes (n = 87 and 45 in the PAD and in the control group, respectively).

### Insulin resistance in study groups

**Figure 1:**
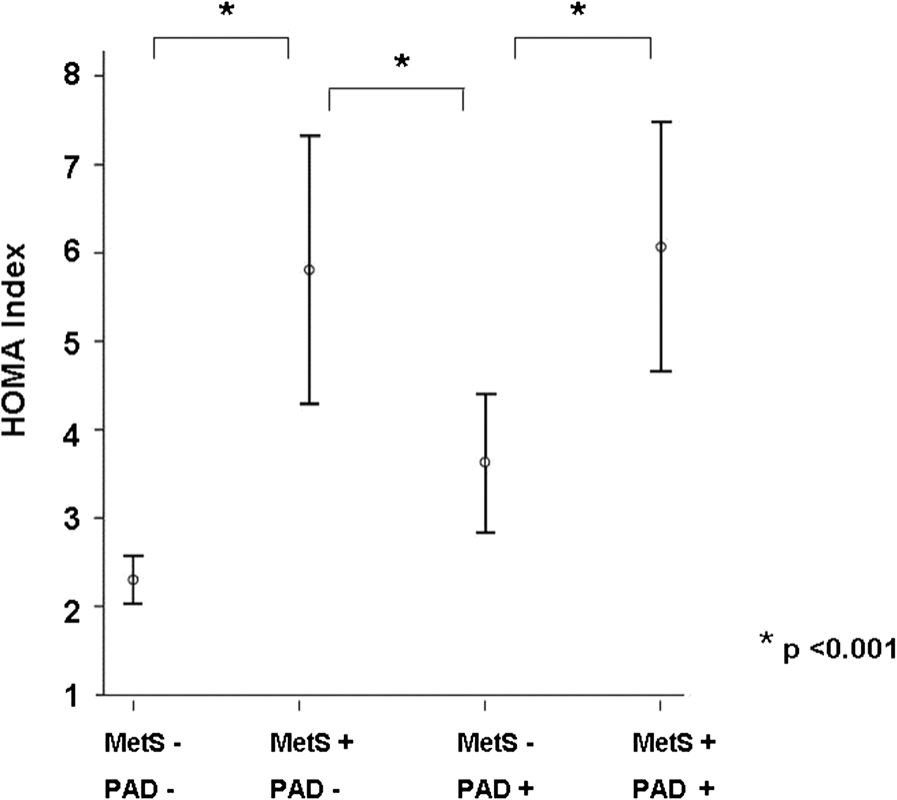
**HOMA insulin resistance scores in subgroups.** Graph shows mean values together with standard deviations. PAD denotes peripheral arterial disease; MetS metabolic syndrome; HOMA homeostasis model assessment.

HOMA-IR scores were higher in MetS patients (NCEP-ATP-III criteria) than in subjects without the MetS (5.9 ± 6.2 vs. 2.9 ± 3.9; p<0.001). However, HOMA IR did not differ significantly between patients with PAD and controls (4.2 ± 5.4 vs. 3.3 ± 4.3; p = 0.124). When both, the presence of the MetS and of PAD was considered (Figure 1), HOMA-IR was significantly higher in patients with the MetS both among those with PAD (6.1 ± 5.7 vs. 3.6 ± 5.2; p <0.001) and among controls (5.8 ± 6.8 vs. 2.3 ± 1.8; p <0.001), whereas it did not differ significantly between patients with PAD and controls among patients with the MetS (5.8 ± 6.8 vs. 6.1 ± 5.7; p = 0.587) nor among those without the MetS (2.3 ± 1.8 vs. 3.6 ± 5.2; p = 0.165) (Table 4).

**Table 4:** HOMA insulin resistance in subgroups

	**PAD +**	**Controls**
**MetS +**	6.1 ± 5.7	5.8 ± 6.8
**MetS -**	3.6 ± 5.2	2.3 ± 1.8

MetS denotes metabolic syndrome.

PAD denotes peripheral arterial disease.

Analysis of covariance (ANCOVA) adjusting for age, gender, smoking, LDL cholesterol, the glomerular filtration rate, antidiabetic medication and alcohol consumption concordantly showed that HOMA insulin resistance was significantly associated with the MetS as diagnosed by ATP-III criteria (F = 12.5; p = <0.001) but not with sonographically proven PAD (F = 0.86; p = 0.670).

### Exclusion of patients with T2DM

In a subgroup analyses, we excluded subjects with T2DM. Still, HOMA insulin resistance scores were significantly higher in patients with MetS than in subjects without MetS among non-diabetic subjects (4.1 ± 3.0 vs. 2.2 ± 1.6; p <0.001) but were again not significantly different between the patients with sonographically proven PAD and controls (2.6 ± 1.9 vs. ± 2.6 ± 2.1; p = 0.644). Also, when we only excluded the patients with diabetes who were on insulin, HOMA insulin resistance scores were significantly higher in patients with the MetS than in subjects without the MetS (5.4 ± 4.0 vs. 2.9 ± 2.3; p <0.001) but were again not significantly different between the patients with sonographically proven PAD and controls (2.7 ± 1.5 vs. 2.6 ± 2.0; p = 0.599).

### IDF definition of the MetS

The prevalence of the MetS according to the IDF definition was 38.7%. Considering both the IDF MetS and the presence of sonographically proven PAD, 129 patients had neither the IDF MetS nor sonographically proven PAD, 68 had the MetS, but not sonographically proven PAD, 123 did not have the MetS but had sonographically proven PAD, and 91 had both, the MetS according to IDF criteria and sonographically proven PAD.

As with the NCEP-ATP-III definition of the MetS, HOMA insulin resistance was significantly higher in patients with the IDF MetS than in subjects who did not have the IDF MetS in the total study population (5.5 ± 5.9 vs. 2.7 ± 3.7; p <0.001) and both among patients with sonographically proven PAD (5.6 ± 5.5 vs.3.2 ± 5.2; p < 0.001) and in the subgroup of control subjects (5.4 ± 6.4 vs. 2.2 ± 1.5; p <0.001), whereas HOMA insulin resistance did not differ significantly between patients with sonographically proven PAD and control subjects both among patients without the IDF MetS (p = 0.442) and among patients who had the MetS according to IDF criteria (p = 0.576).

## Discussion

From our results we conclude that insulin resistance as assessed by the HOMA index is significantly associated with the MetS but is not directly linked to sonographically determined peripheral atherosclerosis.

### Insulin resistance, the metabolic syndrome, and peripheral arterial disease: what this study contributes to the literature

Insulin resistance pathophysiologically is the key feature of the MetS, and correlations between HOMA insulin resistance and the MetS as a clinical entity have been described in numerous previous investigations [9,10,31], whereas the syndrome according to most definitions is diagnosed as a clinical category in the presence of MetS stigmata other than insulin resistance scores. We had published on this important issue previously [8]. Insulin resistance is a complex and multifaceted disorder which is extensively addressed in current biomedical research. Muneyuki et al. [32] for example showed in a small cohort after adjusting for BMI that low serum amylase was associated with decreased basal insulin levels and high insulin resistance. Another investigation by Snoer et al. [33] found that there is an association between insulin resistance, reduced exercise tolerance and reduced coronary flow reserve in heart failure patients. Further to the results of earlier studies [17,34] we demonstrate the association of insulin resistance with the clinical entity of the MetS and with the individual MetS stigmata among patients with PAD, a population of a particular clinical interest.

The key finding of our investigation, however, is that insulin resistance is not associated with sonographically proven peripheral atherosclerosis. Hardly any data are available from the literature on the association between insulin resistance and PAD. Indeed, only two previous investigations, one of them by Pande et al. [35] had addressed this association; however, these studies differed in important aspects from our investigation. Pande et al. investigated the general population and used the ABI instead of ultrasound to diagnose PAD. The mentioned second study by Britton et al. [36] also used the ABI together with a definition of "clinical PAD" based on patient history and physical examination. Theses authors described a weak but significant link between PAD and insulin resistance. However, it is important to consider that the direct visualization of atherosclerosis may reflect other features of the development of PAD than measurement of ABI or other non-visualised PAD features. Thus, both patient selection and perhaps even more importantly the diagnostic modality may have contributed to the divergence of our findings with the work reported by Panda [35] and Britton [36].

Our work is the first report on the association between insulin resistance and PAD using peripheral artery sonography. Most importantly, our study, as evidenced by sample size calculation, was adequately powered to firmly support also a negative finding. Thus, our investigation for the first time firmly establishes that there is no association between insulin resistance and sonographically proven peripheral atherosclerosis.

The possibility should be considered that the high prevalence of smoking observed in our cohort may have overwhelmed the impact of other risk factors. Whatsoever, because a high smoking prevalence is typical for cohorts of PAD patients, the high smoking prevalence in our study population of consecutive, unselected PAD patients reflects what is current clinical reality. As we have published earlier, impaired kidney function is an important indicator of elevated cardiovascular risk [37]; however, our results were confirmed after adjustment for the estimated glomerular filtration rate.

### Strengths and limitations

Important strengths of our study are the direct visualization of peripheral atherosclerosis by sonography, the adequate size of the study population, and the meticulous characterization of study subjects. Because the patients were recruited consecutively, the studied patient sample mirrors the real world scenario of a typical large angiology outpatient clinic. Of course we cannot exclude that this patient sample differs e.g. from PAD patients who are cared for in private practice or who are hospitalized.

We acknowledge the limitation that because of our cross-sectional study design the causality of relationships between parameters cannot be proven; a future study that longitudinally addresses the association of insulin resistance with the progression of sonographically proven atherosclerosis therefore will be of great interest. Further, there are limitations of the HOMA technique to measure insulin resistance. The HOMA index on the one hand is a firmly established measure of insulin resistance which is typically applied in large scale epidemiological studies and therefore guarantees the comparability of our study results with the data from the literature. However, on the other hand HOMA is rarely used for clinical decision making and, more importantly, it should be considered that HOMA insulin resistance scores like all established markers of insulin resistance are based on the estimation of insulin effects on glucose metabolism which of course do not optimally reflect the much broader metabolic consequences of insulin resistance, e.g. for lipid metabolism. Future research aiming at the investigation of insulin resistance over and above the insulin-glucose axis therefore is necessary.

## Conclusions

In conclusion, with respect to HOMA insulin resistance, our study clearly shows the lack of an association between insulin resistance and sonographically proven PAD. Together with our previous observation, that insulin resistance is not associated with coronary atherosclerosis [13], our findings suggest that it may play a more important role in the thrombotic features of atherothrombotic disease (which eventually precipitate the clinical cardiovascular event) than in the development of atherosclerosis (which is visualized by sonography or angiography).

## Abbreviations

MetS: Metabolic Syndrome; CAD: Coronary artery disease; PAD: Peripheral arterial disease; T2DM: Type 2 Diabetes; HDL: High Density Lipoprotein; LDL: Low Density Lipoprotein; IDF: International Diabetes Federation; ABI: Ankle Brachial Index; BMI: Body Mass Index; HOMA: Homeostasis Model Assessment; NCEP-ATPIII: National Cholesterol Education Programme ATP-III.

## Competing interests

The authors declare that they have no conflict of interest.

## Authors’ contributions

AV contributed to the concept and design of the study; to the collection of data; to analysis and interpretation of data; and to drafting the article. CHS contributed to the concept and design of the study; to the collection of data; to the analysis and interpretation of data and to revising the manuscript for important intellectual content. PR contributed to the collection of data; to analysis and interpretation of data; and to revising the manuscript for important intellectual content. HD contributed to the concept and design of the study; to analysis and interpretation of data; and to drafting the article. AV and CHS contributed equally to this work. All authors read and approved the final manuscript.
